# Work-time arrangements, career progression, and job satisfaction among physicians in Germany: a cross-sectional survey

**DOI:** 10.1186/s12913-026-15756-9

**Published:** 2026-10-21

**Authors:** Vogel Carolina, Stangl Dorina, Habel Silke, Histing Tina, Büchin Petra

**Affiliations:** 1 Department of Trauma and Reconstructive Surgery, University Tübingen, BG Trauma Center, Tübingen, Germany; 2 Department of Cardiology and Pulmonology, Fürth Hospital (Klinikum Fürth), Fürth, Germany; 3 Department of Trauma and Orthopaedic Surgery, Offenburg Trauma Center, Ortenau Clinic Group, Offenburg, Germany; 4 Center for Spine Surgery and Spine Therapy, Karl Olga Hospital, Stuttgart, Germany

**Keywords:** Work schedule tolerance, Physicians, Career mobility, Job satisfaction, Gender equity

## Abstract

**Background:**

Demographic change and evolving expectations regarding work–life balance are increasingly challenging the medical workforce in Germany. Work-time arrangements play a key role in physician job satisfaction, well-being, and career development. However, part-time and alternative working models remain unevenly implemented and are frequently perceived as limiting career opportunities. This study characterized current work-time arrangements among physicians in Germany and examined satisfaction, employer support, colleague acceptance, perceived career opportunities, barriers to reducing working hours, and work–family balance.

**Methods:**

This cross-sectional study is based on an anonymous digital survey conducted by the German Medical Women’s Association between September 2022 and July 2023. Physicians across medical specialties and career stages were invited to participate. The questionnaire assessed sociodemographic and professional characteristics, work-time arrangements (full-time, part-time, and alternative models), satisfaction with current arrangements, employer support, and perceived career opportunities.

**Results:**

Of the 898 respondents, 90.9% were women and 46.2% were resident physicians. Full-time employment was the most common arrangement (56.8%). Physicians working in non-full-time arrangements were more frequently satisfied with their work-time arrangement than those working full-time (52.8% vs. 45.3%; *p* = 0.020). Hospital-based physicians were less likely to be satisfied with their work-time arrangement (aOR 0.33) or to receive support from their employer in implementing alternative work-time arrangements (aOR 0.24). They were also less likely to report acceptance of alternative work-time arrangements among colleagues (aOR 0.21) and equal career opportunities under such arrangements (aOR 0.31), while being more likely to report difficulties balancing work and family responsibilities (aOR 2.44; all *p* < 0.001). Compared with physicians in leadership positions, resident physicians and board-certified physicians without leadership responsibility reported lower satisfaction and lower perceived equal career opportunities, while reporting greater difficulty balancing work and family responsibilities (all *p* < 0.001). Fear of negative career consequences was the most frequently reported reason for not reducing working hours (58.2%), and approximately two thirds of respondents perceived unequal career opportunities for colleagues using alternative work-time arrangements.

**Conclusions:**

Although full-time employment remained the most common arrangement, respondents not working full-time reported greater satisfaction. Part-time and alternative work-time-arrangements are widely perceived as career-limiting. Addressing structural barriers and ensuring equal career opportunities for physicians who require or prefer family-friendly working conditions are essential for physician well-being, workforce retention, and the long-term resilience of healthcare systems.

**Supplementary Information:**

The online version contains supplementary material available at https://doi.org/10.1186/s12913-026-15756-9.

## Background

Demographic change represents a major societal challenge in Germany and increasingly affects the medical workforce. In particular, the age-related retirement of the baby boomer generation is expected to further exacerbate the existing shortage of physicians across medical specialties. This development has intensified discussions on the recruitment and long-term retention of early-career physicians. At the same time, multiple surveys have demonstrated a pronounced mismatch between physicians’ working realities and the expectations of medical students regarding their future professional lives.

At the end of 2022, 421,303 physicians were professionally active in Germany. Of these, 51.6% worked in inpatient care, 39.3% in ambulatory care, and 9.1% in public authorities, corporations, or other professional settings. Women accounted for 49.0% of the professionally active physician workforce [1].

Work-time arrangements have a substantial impact on job satisfaction, work–life balance, and conflicts between professional and private life [2]. Currently, approximately 64% of physicians in Germany work full-time. Among these, 71% report weekly working hours exceeding 49 h, and 24% report working more than 60 h per week [3]. However, most physicians report a preferred weekly work-time of 40–48 h, with 37% indicating a wish to reduce their working hours [3]. Although part-time employment typically corresponds to 29–39 working hours per week, more than half of part-time physicians still report working more than 40 h weekly [3]. High workload and work intensification contribute to the consideration of leaving the medical profession in up to 80% of physicians, while 72% report a discrepancy between their professional reality and their personal professional standards [3]. Particularly among surgeons, around 65% perceive a substantial risk of burnout, with overtime and long working hours representing key risk factors for psychosocial health impairments [4].

In addition to clinical duties, administrative work represents a substantial burden. Physicians report spending an average of approximately three hours per day on administrative tasks. Moreover, 63% indicate a preference for performing at least part of their work remotely [3]. Against this background, part-time and flexible work-time arrangements are gaining relevance even in traditionally full-time-oriented specialties such as orthopedics and trauma surgery, particularly among residents [5].

The changing expectations of the next generation of physicians are further underlined by the German Medical Students’ Career Monitoring Survey conducted in 2022. In this survey, 81% of medical students rated flexible working hours, 83% regulated working hours, and 93% the compatibility of family and professional life as important or very important factors for their future careers [6]. In Germany, female medical students have constituted the majority since 1998 and currently represent approximately 70% of the medical student population. They place particular emphasis on family life and leisure time, with 88% rating these aspects as highly relevant [6].

To ensure sustainable healthcare delivery in clinical practice, research, and teaching, structural approaches are needed that systematically address the expectations and needs of the next generation of physicians. In this context, the development of family-friendly and flexible working conditions plays a central role, particularly in light of changing expectations regarding work–life balance, flexibility, and career sustainability. Despite the high relevance of work-time arrangements for workforce retention and job satisfaction, their implementation remains limited, and concerns regarding potential career disadvantages and organizational feasibility persist.

Against this background, the German Medical Women’s Association (Deutscher Ärztinnenbund) conducted the survey *“How family-friendly are working conditions in medicine?”*. The present study aimed to characterize current work-time arrangements among physicians in Germany, assess satisfaction with these arrangements, examine perceived employer support and acceptance, and explore barriers, perceived career implications, and work–family balance across relevant sociodemographic and professional groups.

## Methods

### Study design

The present analysis is based on a digital cross-sectional survey entitled “How family-friendly are working conditions in medicine?”, conducted by the German Medical Women’s Association (Deutscher Ärztinnenbund, DÄB) between 6 September 2022 and 1 July 2023.

### Eligibility criteria

Eligible participants were physicians working in Germany across all medical specialties and career stages. The present analysis included all respondents.

### Survey

The survey was developed by an expert group from the German Medical Women’s Association and covered two broad domains: work–family compatibility as well as working and career conditions among physicians in Germany. The present analysis focuses on items assessing employment setting, work-time arrangements, satisfaction with these arrangements, employer support, perceived acceptance, barriers to reducing working hours, perceived career implications, and work–family balance.

Before its distribution, the questionnaire was internally pretested by members of the physician expert group to assess the comprehensibility of the items, the clarity of the wording, and the technical functionality of the survey. Feedback obtained during this process was used to refine the final version of the questionnaire. However, no formal pilot study or psychometric validation was performed.

The survey was disseminated via the DÄB email mailing list and through professional and social networks. Participation was voluntary and anonymous.

All 898 respondents who participated in the survey were included in the present analysis. Because the number of individuals who received or encountered the survey invitation was unknown, a response rate could not be calculated.

The online questionnaire comprised 34 items (see additional file 1 for the full questionnaire) covering sociodemographic characteristics such as age and gender, professional characteristics including medical specialty, career stage, and employment sector, and current work-time arrangements.

Gender was recorded as woman, man, or diverse. The one participant identifying as diverse was included in descriptive analyses but excluded from gender-stratified analyses and multivariable models because the group size did not permit a separate statistical analysis.

Age was assessed using predefined categorical response options (< 25, 25–30, 30–35, 35–40, 40–45, > 45 years). Although category boundaries were not strictly mutually exclusive, each participant was required to select a single response option, ensuring unique assignment to one age category.

Current work-time arrangement was derived from fixed-choice and free-text responses and recoded into mutually exclusive categories using a hierarchical classification rule. When full-time employment was explicitly stated, participants were classified as working full-time irrespective of any additional arrangement mentioned. When part-time employment was stated together with another arrangement, participants were classified as working part-time. For all remaining free-text responses, the first arrangement mentioned was used as the primary work-time arrangement. For the main analyses, part-time employment, alternative primary work-time arrangements, and parental leave were combined as non-full-time employment. Classification as full-time or part-time was based on participants’ self-reported employment status; no standardized threshold based on weekly working hours was applied. Alternative arrangements included remote work, flexitime, job-sharing, and other participant-specified arrangements. These arrangements were not defined using standardized operational criteria and therefore reflected the participants’ own descriptions.

Medical specialties were analyzed both as individual categories and as a dichotomized variable comprising operative and non-operative specialties. All individual specialties with ≥ 1% are presented; specialties reported by fewer than 1% of participants were combined into an “other” category for descriptive analysis. Regarding the dichotomized variable, the operative group included general surgery (*n* = 175), orthopedics and trauma surgery (*n* = 69), gynecology and obstetrics (*n* = 99), urology (*n* = 21), neurosurgery (*n* = 16), pediatric surgery (*n* = 2), cardiac surgery (*n* = 1), oral and maxillofacial surgery (*n* = 6), otolaryngology (*n* = 10), plastic surgery (*n* = 1), and anesthesiology (*n* = 79). All remaining specialties were classified as non-operative.

Employment sector was dichotomized as hospital versus non-hospital employment. The non-hospital category comprised outpatient medical practice, research and teaching, public health, industry, and other employment settings. Career stage was analyzed using three categories: resident physicians; board-certified physicians without leadership responsibility, comprising board-certified physicians and board-certified senior physicians; and physicians with leadership responsibility, comprising lead senior physicians and chief physicians. Parental status was dichotomized as participants without children versus parents. In the questionnaire, five-point Likert-scale items were coded in ascending order, with higher categories indicating stronger agreement (strongly disagree, disagree, neither agree nor disagree, agree, and strongly agree). For the question assessing reasons for deciding against a desired reduction in working hours, multiple responses were permitted. Each response option was analyzed as a binary variable indicating whether the respective reason had been selected.

### Ethics

This study was conducted in accordance with the principles of the Declaration of Helsinki and adhered to standards of Good Scientific Practice. Ethical approval was not required, as the study was based on an anonymous and voluntary online survey that did not involve patients, medical interventions, or the collection of identifiable personal data. No directly identifying information was collected, and data handling complied with the General Data Protection Regulation [7]. Participants were informed about the purpose of the survey at the beginning, and participation was entirely voluntary. Completion of the questionnaire was considered to constitute informed consent.

### Data collection and statistical analysis

Data collection was performed using the SurveyMonkey^®^ platform. Statistical analyses and data visualization were conducted using IBM SPSS Statistics (version 30.0.0.0) and Microsoft Excel (version 16.112). No formal a priori sample-size calculation was performed. The sample size was determined by the number of eligible responses received during the predefined survey period. Because the number of individuals who received or encountered the survey invitation was unknown, a response rate could not be calculated. Categorical variables are presented as absolute numbers and relative frequencies. Missing values were not imputed. Analyses were performed using all available responses for each variable. Percentages were calculated based on valid responses. For multiple-response questions on reasons for not reducing working hours, percentages are based on respondents who answered the question and may exceed 100%. Associations between categorical variables were tested using Pearson’s chi-square tests. Likert-scale outcomes were compared using the full five-point response scale rather than dichotomized categories. Five key outcomes were analyzed using multivariable ordinal logistic regression: satisfaction with work-time arrangement, perceived employer support for alternative arrangements, acceptance of colleagues using such arrangements, perceived equal career opportunities, and difficulty balancing work and family life. All models included the same predictors: gender (women vs. men), work-time arrangement (full-time vs. non-full-time), employment sector (hospital vs. non-hospital), specialty (non-operative vs. operative), career stage (residents and board-certified physicians without leadership vs. physicians in leadership roles), and parental status (no children vs. parents). These variables were chosen because of their relevance to work conditions, career development, and work–life balance. Reference groups were men, non-full-time employment, non-hospital employment, operative specialties, physicians in leadership positions, and parents. Results are shown as adjusted odds ratios (aORs) with 95% confidence intervals (CIs). An aOR > 1 indicates higher odds of a higher response category. The proportional-odds assumption was checked using the test of parallel lines and was met in all models. Model fit was assessed with likelihood-ratio chi-square tests. All analyses used complete cases, with 897 participants included in each model. All tests were two-sided. Because the analyses were exploratory, p-values were not adjusted for multiple testing and should be interpreted accordingly. A p-value of ≤ 0.05 was considered statistically significant.

## Results

A total of 898 physicians participated in the survey. Of these, 90.9% were women, 9.0% were men, and 0.1% identified as diverse (Table 1).

### Work-time arrangements

The reported work-time arrangements are presented in Table 1. Women worked in non-full-time arrangements more frequently than men (44.7% vs. 28.4%; *p* = 0.005; Table 1).

Among respondents, 63.9% were parents (Table 1). Parents worked in non-full-time arrangements substantially more frequently than participants without children (59.1% vs. 15.1%; *p* < 0.001; Table 1). Nearly half of the respondents were resident physicians (46.2%; Table 1). The distribution of work-time arrangements differed significantly across career stages, as shown in Table 1 (*p* < 0.001).Medical specialties are shown in Table 1, with operative specialties accounted for 53.3% of the total cohort. The distribution of full-time and non-full-time arrangements did not differ between operative and non-operative specialties (*p* = 0.791; Table 1). The majority of participants worked in the hospital sector (83.6%; Table 1). Physicians working in non-hospital settings were significantly more frequently employed in non-full-time arrangements than those working in the hospital sector (59.2% vs. 40.1%; *p* < 0.001; Table 1). 56.9% reported having no access to employer-provided childcare, whereas 25.6% had access to childcare compatible with their working hours (Table 1). Of the 662 participants who responded to the optional question about using hospital-provided childcare, 73.4% indicated that they would use such a service. Willingness to use employer-provided childcare did not differ significantly by gender (*p* = 0.192), career stage (*p* = 0.559), or specialty group (non-operative vs. operative; *p* = 0.136). Physicians without children were significantly more likely to indicate that they would use this service than physicians with children (91.0% vs. 70.3%; *p* < 0.001).

**Table 1:** Participant characteristics according to current work-time arrangement.Data are presented as N and percentages. Percentages in the Total column describe the distribution within the respective valid sample, whereas percentages in the Full-time and Non-full-time columns are row percentages

				Work-time arrangement				
		Total		Full-time		Non-full-time		*p* value
		N	%	N	%	N	%	
Full cohort		898	100	510	56.8	388	43.2	
Gender	*Women*	816	90.9	451	55.3	365	44.7	*0.005*
	*Men*	81	9.0	58	71.6	23	28.4	
	Diverse*	1	0.1	1	100.0	0	0.0	
Parental status	*No*	324	36.1	275	84.9	49	15.1	*< 0.001*
	*Yes*	574	63.9	235	40.9	339	59.1	
Single-parent**	*No*	538	95.4	222	41.3	316	58.7	*0.501*
	*Yes*	26	4.6	9	34.6	17	65.4	
Employment/ career status	*Resident physician*	415	46.2	271	65.3	144	34.7	*< 0.001*
	*Board-certified physicians*	247	27.5	96	38.9	151	61.1	
	*Board-certified senior physicians*	126	14.0	54	42.9	72	57.1	
	*Lead senior physician*	61	6.8	44	72.1	17	27.9	
	*Lead chief physician*	49	5.5	45	91.8	4	8.2	
Specialty group	*Non-operative*	419	46.7	236	56.3	183	43.7	*0.791*
	*Operative*	479	53.3	274	57.2	205	42.8	
Medical specialty	*Surgical specialties (all)*	291	32.4	177	60.8	114	39.2	*0.186*
	*Internal medicine*	181	20.2	112	61.9	69	38.1	
	*Gynecology and obstetrics*	99	11.0	45	45.5	54	54.5	
	*Anesthesiology*	79	8.8	42	53.2	37	46.8	
	*Neurology*	38	4.2	24	63.2	14	36.8	
	*Pediatrics*	38	4.2	19	50.0	19	50.0	
	*General practice*	36	4.0	18	50.0	18	50.0	
	*Radiology*	35	3.9	17	48.6	18	51.4	
	*Psychiatry*	20	2.2	11	55.0	9	45.0	
	*Other*	81	9.0	45	55.6	36	44.4	
Hospital vs. non-hospital	*Hospital*	751	83.6	450	59.9	301	40.1	*< 0.001*
	*Non-hospital*	147	16.4	60	40.8	87	59.2	
Availability of employer-provided childcare***	*No*	375	56.9	176	46.9	199	53.1	*0.586*
	*Yes*,* compatible with working hours*	169	25.6	79	46.7	90	53.3	
	*Yes*,* not compatible with working hours*	115	17.5	60	52.2	55	47.8	

*** Data on the availability of employer-provided childcare were available for 659 participants

* The one participant identifying as diverse was included in the descriptive results but excluded from the inferential gender comparison because the group size did not permit a separate statistical analysis

** Single-parent status was analyzed among participants reporting parenthood.Data were available for 564 of 574 parents

**Fig. 1:**
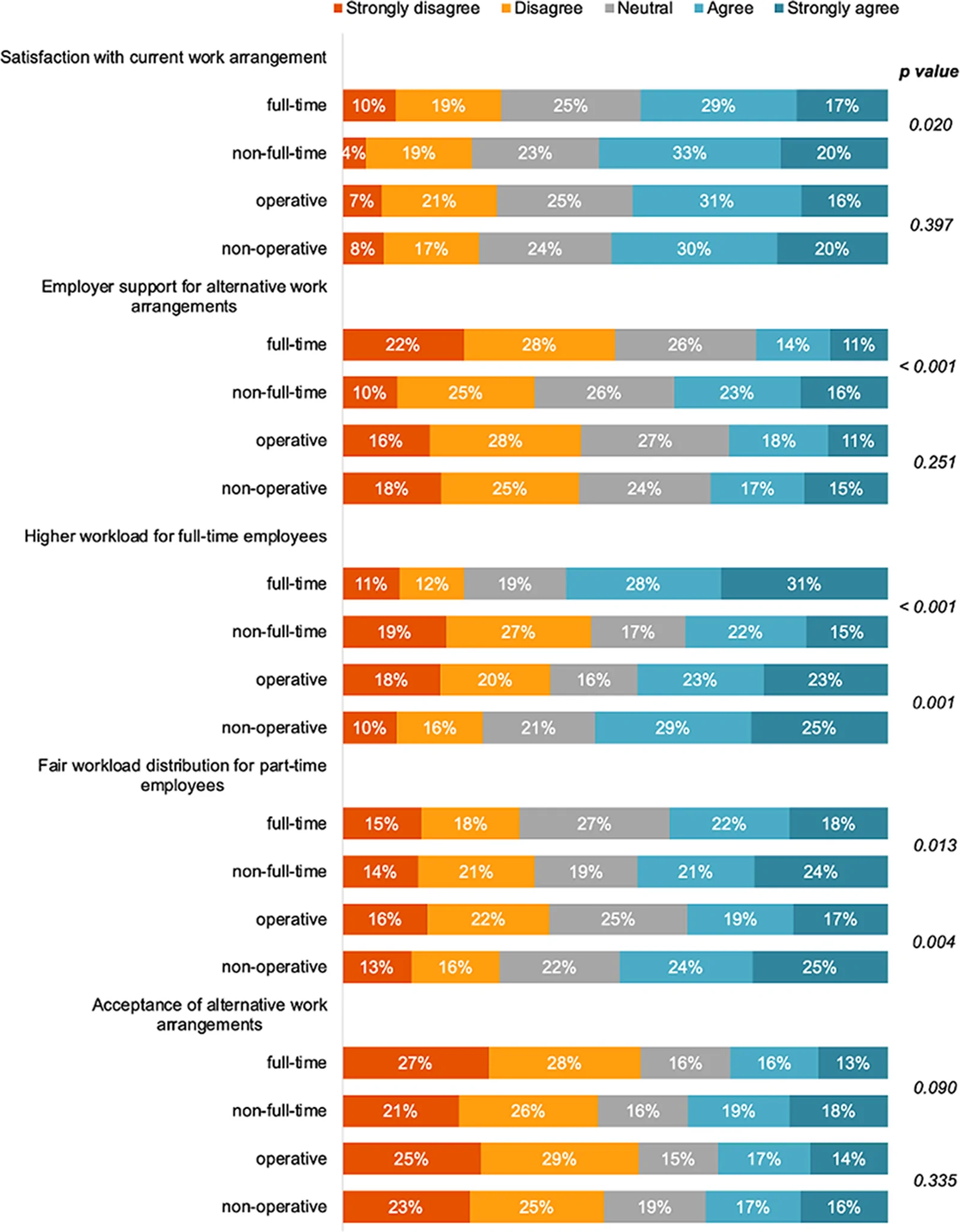
Satisfaction with current work-time arrangements and perceptions of alternative work-time arrangements. Responses are presented according to current work-time arrangement (full-time versus non-full-time) and specialty group (operative versus non-operative). Stacked bars show the percentage of valid responses in each of the five Likert categories. Values below 4% were omitted from the labels to improve readability. Group comparisons were based on the complete five-category response distributions using Pearson’s chi-square tests

Full-time physicians were less frequently satisfied with their current work-time arrangement than physicians not working full-time (45.3% vs. 52.8% agreeing or strongly agreeing; *p* = 0.020; Fig. 1). Satisfaction did not differ significantly between physicians working in operative and non-operative specialties (46.8% vs. 50.6%; *p* = 0.397; Fig. 1). Employer support for alternative work-time arrangements was reported less frequently by full-time physicians than by physicians not working full-time (24.1% vs. 39.2% agreeing or strongly agreeing; *p* < 0.001). No significant difference was observed between operative and non-operative specialties (29.0% vs. 32.5%; *p* = 0.251; Fig. 1).

Participants working full-time agreed or strongly agreed more frequently that alternative work-time arrangements resulted in a higher workload for full-time staff than participants not working full-time (59.0% vs. 37.1%; *p* < 0.001; Fig. 1). Across specialty groups, this perception was also more common among participants in non-operative than operative specialties (53.7% vs. 45.9%; *p* = 0.001; Fig. 1).

Perceptions of whether duties, including weekend duties, were distributed fairly when colleagues worked part-time differed by both employment status and specialty. Physicians not working full-time more frequently agreed or strongly agreed that the distribution was fair than full-time physicians (45.9% vs. 40.0%; *p* = 0.013). Agreement was also more frequent among physicians in non-operative than operative specialties (49.2% vs. 36.7%; *p* = 0.004; Fig. 1).

More than half of full-time physicians and physicians in operative specialties disagreed or strongly disagreed that colleagues using alternative work-time arrangements were as accepted as full-time staff in their departments (54.7% and 54.3%; Fig. 1). However, the complete response distributions did not differ significantly between full-time and non-full-time physicians (*p* = 0.090) or between operative and non-operative specialties (*p* = 0.335; Fig. 1).

In the adjusted ordinal regression analyses (aOR), full-time employment was associated with lower odds of reporting greater satisfaction with the current work-time arrangement (aOR 0.65; *p* = 0.002), greater employer support for alternative work-time arrangements (aOR 0.49; *p* < 0.001), and greater acceptance of colleagues using such arrangements (aOR 0.58; *p* < 0.001; Table 2). Hospital employment was likewise associated with lower odds across all three outcomes, with aORs ranging from 0.21 to 0.33 (all *p* < 0.001; Table 2). Compared with physicians in leadership positions, both resident physicians and board-certified physicians without leadership responsibility reported lower satisfaction, less employer support, and lower acceptance of colleagues in alternative work-time arrangements (all *p* < 0.001; Table 2). Women reported greater satisfaction with their work-time arrangement than men (aOR 1.58; *p* = 0.029), whereas no gender differences were found for perceived employer support or acceptance of colleagues in alternative work-time arrangements. Participants without children reported greater satisfaction (aOR 1.56; *p* = 0.002) and acceptance of colleagues in alternative work-time arrangements (aOR 1.47; *p* = 0.006) than parents. Specialty group was not independently associated with any of the three outcomes (Table 2).

**Table 2:** Multivariable ordinal logistic regression analyses of work-time arrangements

a) Satisfaction with current work-time arrangement		
Predictor	aOR (95% CI)	*p* value
Women vs. men	1.58 (1.05–2.39)	*0.029*
Full-time vs. non-full-time	0.65 (0.49–0.85)	*0.002*
Hospital vs. non-hospital	0.33 (0.24–0.47)	*< 0.001*
Non-operative vs. operative	1.01 (0.79–1.29)	*0.932*
Resident physicians vs. physicians in leadership positions	0.32 (0.22–0.48)	*< 0.001*
Board-certified physicians without leadership vs. physicians in leadership positions	0.38 (0.26–0.58)	*< 0.001*
No children vs. parents	1.56 (1.18–2.05)	*0.002*
**b) Employer support for alternative work-time arrangements**		
**Predictor**	**aOR (95% CI)**	***p value***
Women vs. men	1.42 (0.94–2.14)	*0.100*
Full-time vs. non-full-time	0.49 (0.37–0.64)	*< 0.001*
Hospital vs. non-hospital	0.24 (0.17–0.34)	*< 0.001*
Non-operative vs. operative	0.85 (0.67–1.09)	*0.195*
Resident physicians vs. physicians in leadership positions	0.51 (0.35–0.75)	*< 0.001*
Board-certified physicians without leadership vs. physicians in leadership positions	0.42 (0.28–0.63)	*< 0.001*
No children vs. parents	1.10 (0.84–1.45)	*0.494*
**c) Acceptance of colleagues in alternative work-time arrangements**		
**Predictor**	**aOR (95% CI)**	***p value***
Women vs. men	1.11 (0.73–1.67)	*0.638*
Full-time vs. non-full-time	0.58 (0.44–0.77)	*< 0.001*
Hospital vs. non-hospital	0.21 (0.15–0.30)	*< 0.001*
Non-operative vs. operative	0.93 (0.73–1.19)	*0.557*
Resident physicians vs. physicians in leadership positions	0.34 (0.23–0.51)	*< 0.001*
Board-certified physicians without leadership vs. physicians in leadership positions	0.27 (0.18–0.41)	*< 0.001*
No children vs. parents	1.47 (1.12–1.94)	*0.006*

All three multivariable models included 897 participants. All predictors shown were included simultaneously. An aOR > 1 indicates higher odds of reporting a higher response category. Reference categories were men, non-full-time employment, non-hospital setting, operative specialty, physicians in leadership positions, and parents. All overall models were statistically significant (*p* < 0.001)

Fear of negative career consequences was the most frequently reported reason for deciding against a desired reduction in working hours (58.2%; Table 3). Other frequently reported reasons are shown in Table 3.

**Table 3:** Reasons for deciding against a desired reduction in working hours, overall, by gender and by specialty

*N*	Total	Women	Men	*p* valueᵃ	Non-op	Op	*p* valueᵇ
	845	770	74		389	456	
**Reason** (N (%))							
Fear of negative career consequences	492 (58.2%)	444 (57.7%)	47 (63.5%)	*0.330*	204 (52.4%)	288 (63.2%)	*0.002*
Staff shortage	364 (43.1%)	328 (42.6%)	36 (48.6%)	*0.315*	178 (45.8%)	186 (40.8%)	*0.146*
Employer does not accept alternative work-time arrangements	283 (33.5%)	261 (33.9%)	22 (29.7%)	*0.468*	147 (37.8%)	136 (29.8%)	*0.014*
Competitive pressure	157 (18.6%)	144 (18.7%)	13 (17.6%)	*0.811*	45 (11.6%)	112 (24.6%)	*< 0.001*
Concern about others’ opinions	84 (9.9%)	79 (10.3%)	5 (6.8%)	*0.336*	37 (9.5%)	47 (10.3%)	*0.700*

Data were available for 845 participants. Multiple responses were permitted. Percentages refer to respondents within each group and therefore sum to more than 100%. The total sample included one participant identifying as diverse, who was excluded from the gender-stratified analysis. ᵃ Comparison between women and men. ᵇ Comparison between non-operative (non-op) and operative (op) specialties. P values were calculated using Pearson’s chi-square tests

**Fig. 2:**
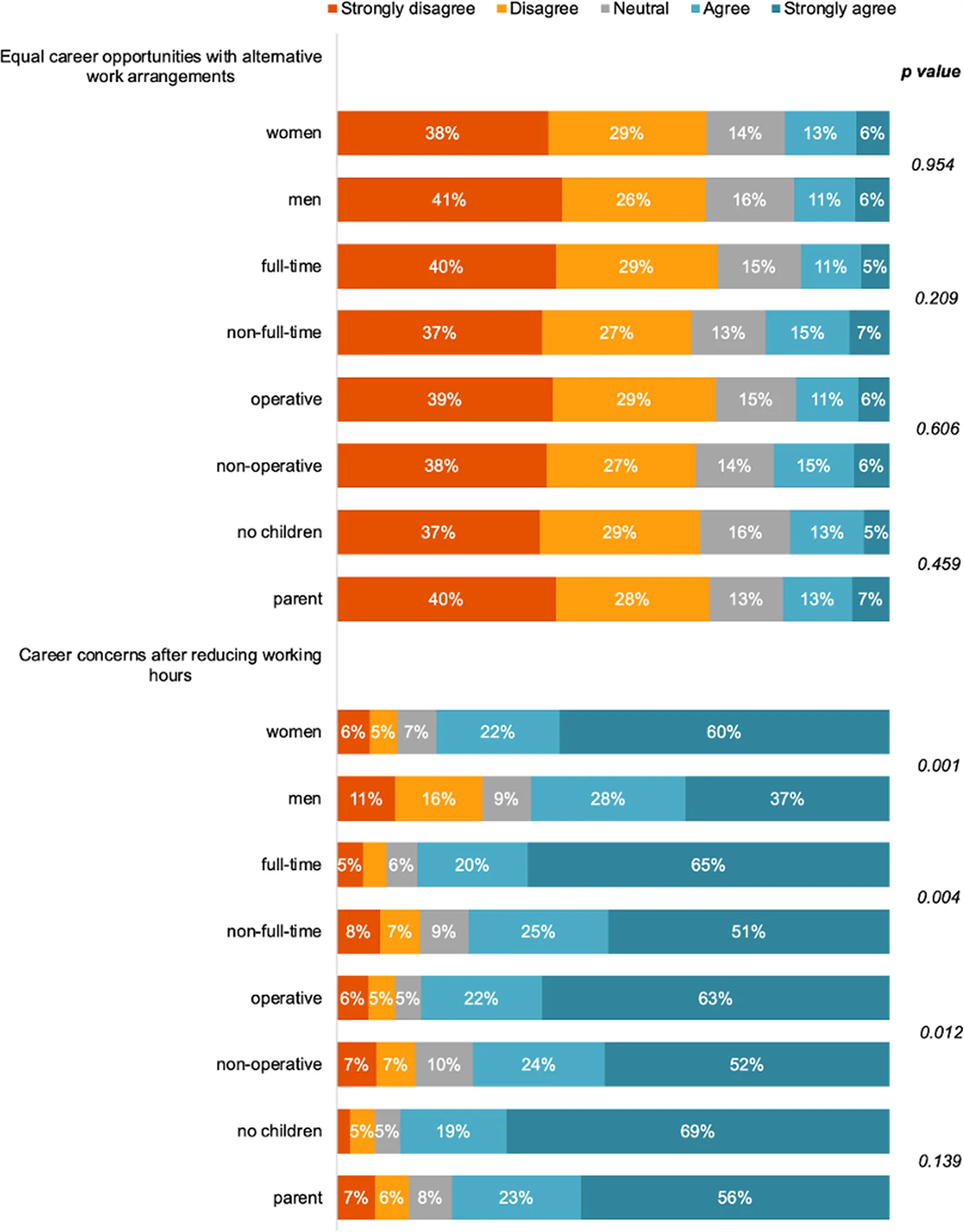
Perceived career implications of alternative work-time arrangements. Responses are presented according to gender (women vs. men), current work-time arrangement (full-time versus non-full-time), specialty group (operative versus non-operative) and parental status (no children vs. parents). Stacked bars show the percentage of valid responses in each of the five Likert categories. Values below 4% were omitted from the labels to improve readability. Group comparisons were based on the complete five-category response distributions using Pearson’s chi-square tests

Approximately two-thirds of both women and men disagreed or strongly disagreed that colleagues using alternative work-time arrangements had the same career opportunities as full-time staff (66.8% and 66.7%; Fig. 2). No significant differences in the complete response distributions were observed by gender (*p* = 0.954), employment status (*p* = 0.209), operative versus non-operative specialty (*p* = 0.606), or parental status (*p* = 0.459; Fig. 2).

Furthermore, 82.0% of women agreed or strongly agreed that they were concerned about negative career consequences if they reduced their working hours, compared with 64.9% of men (*p* = 0.001; Fig. 2). Full-time physicians also reported such concerns more frequently than physicians not working full-time (85.5% vs. 76.1%; *p* = 0.004; Fig. 2). Similarly, agreement was more frequent among physicians working in operative than non-operative specialties (84.8% vs. 75.4%; *p* = 0.012; Fig. 2). There was no difference between physicians without children and parents (*p* = 0.139; Fig. 2).

In the aOR analysis, full-time physicians had lower odds of perceiving equal career opportunities for colleagues using alternative work-time arrangements than non-full-time physicians (aOR 0.69; *p* = 0.009; Table 4). Hospital employment was also associated with substantially lower odds of perceiving equal career opportunities (aOR 0.31; *p* < 0.001; Table 4). Compared with physicians in leadership positions, the odds were lower among both resident physicians (aOR 0.41; *p* < 0.001; Table 4) and board-certified physicians without leadership responsibility (aOR 0.31; *p* < 0.001; Table 4). No independent associations were observed for gender, specialty group, or parental status (Table 4).

**Table 4:** Multivariable ordinal logistic regression analyses of equal career opportunities with alternative work-time arrangements

Predictor	aOR (95% CI)	*p* value
Women vs. men	0.90 (0.59–1.37)	*0.623*
Full-time vs. non-full-time	0.69 (0.52–0.91)	*0.009*
Hospital vs. non-hospital	0.31 (0.22–0.44)	*< 0.001*
Non-operative vs. operative	0.90 (0.70–1.15)	*0.402*
Resident physicians vs. physicians in leadership positions	0.41 (0.28–0.60)	*< 0.001*
Board-certified physicians without leadership vs. physicians in leadership positions	0.31 (0.20–0.46)	*< 0.001*
No children vs. parents	1.32 (1.00–1.75)	*0.052*

The multivariable model included 897 participants. All predictors shown were included simultaneously.An aOR > 1 indicates higher odds of reporting a higher response category. Reference categories were men, non-full-time employment, non-hospital setting, operative specialty, physicians in leadership positions, and parents. The model was statistically significant (*p* < 0.001)

### Work-family balance

**Fig. 3:**
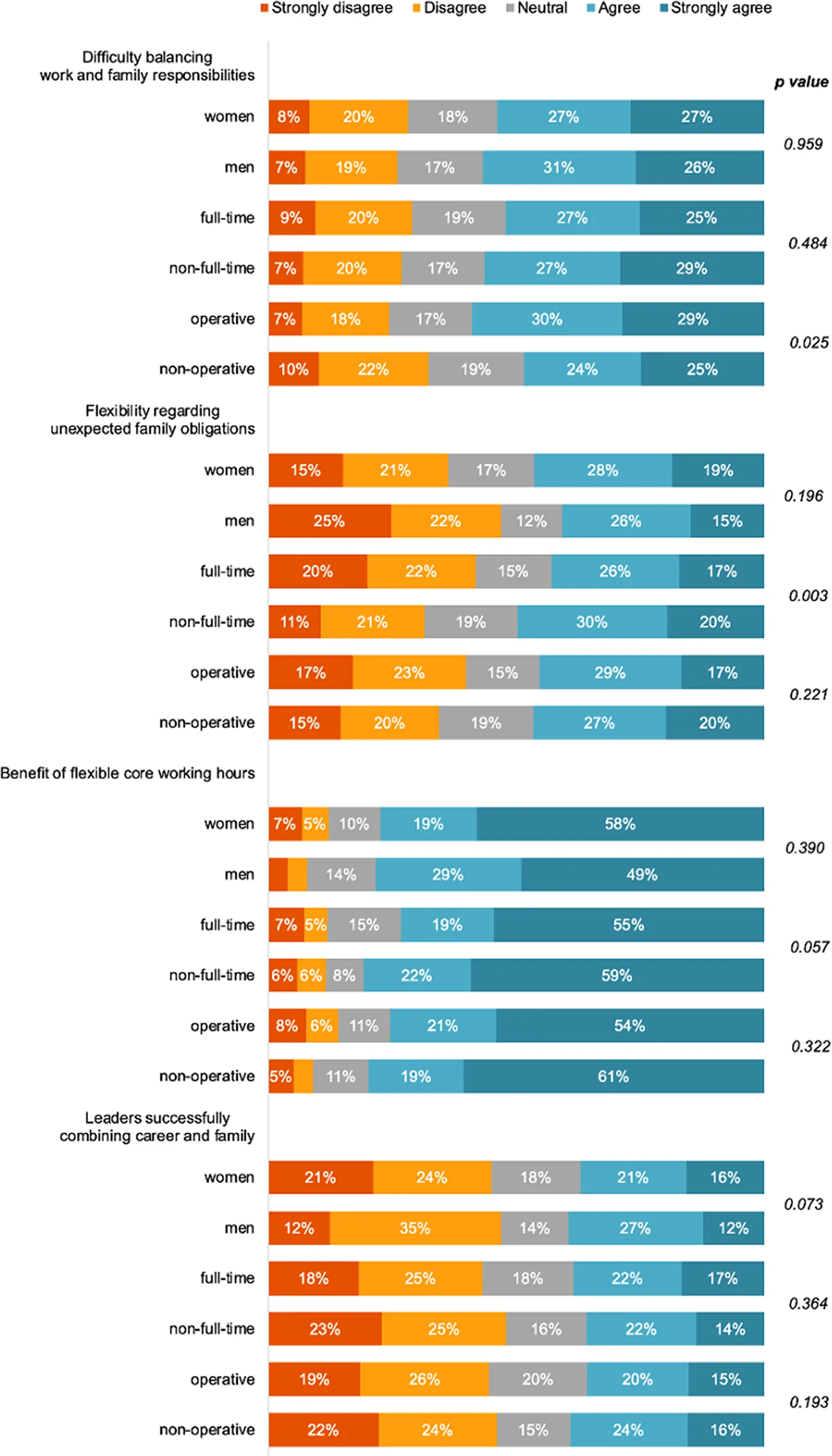
Work–family balance and family-friendly workplace conditions. Responses are presented according to gender (women vs. men), current work-time arrangement (full-time versus non-full-time) and specialty group (operative versus non-operative). Stacked bars show the percentage of valid responses in each of the five Likert categories. Values below 4% were omitted from the labels to improve readability.Group comparisons were based on the complete five-category response distributions using Pearson’s chi-square tests

Women and men reported similar difficulties in balancing work and family responsibilities, with 53.8% and 56.8%, respectively, agreeing or strongly agreeing that this represented a current problem (*p* = 0.959; Fig. 3). The responses also did not differ significantly between full-time and non-full-time physicians (52.2% vs. 56.4% agreeing or strongly agreeing; *p* = 0.484; Fig. 3). Physicians working in operative specialties, however, more frequently agreed or strongly agreed that balancing work and family responsibilities was difficult than those in non-operative specialties (58.9% vs. 48.4%; *p* = 0.025; Fig. 3). However, this difference was attenuated in the aOR analysis (Table 5).

Perceived departmental flexibility in responding to unexpected family obligations did not differ significantly between women and men (46.4% vs. 40.7% agreeing or strongly agreeing; *p* = 0.196; Fig. 3). Participants not working full-time reported such flexibility more frequently than full-time participants (49.7% vs. 42.9%; *p* = 0.003; Fig. 3). No significant difference was observed between operative and non-operative specialties (45.3% vs. 46.5%; *p* = 0.221; Fig. 3).

More than three-quarters of women and men agreed or strongly agreed that greater flexibility regarding core working hours would make their lives easier (77.4% and 78.4%; *p* = 0.390; Fig. 3). Agreement was also high among full-time and non-full-time physicians (73.3% and 80.9%; Fig. 3), although the difference did not reach statistical significance (*p* = 0.057). No significant difference was observed between operative and non-operative specialties (75.4% vs. 79.8%; *p* = 0.322; Fig. 3).

Slightly more than one-third of respondents agreed or strongly agreed that physicians in leadership positions in their professional environment successfully combined career and family (Fig. 3). Agreement was reported by 37.1% of women and 39.5% of men (*p* = 0.073), 38.4% of full-time and 35.8% of non-full-time physicians (*p* = 0.364), and 35.7% of physicians in operative and 39.1% in non-operative specialties (*p* = 0.193; Fig. 3).

The option for pregnant employees to continue working in alternative roles, such as remote work, was reported by 40.0% of women and 28.4% of men (*p* = 0.083; Fig. 3). Agreement did not differ significantly between full-time and non-full-time physicians (36.1% vs. 42.5%; *p* = 0.282) or between operative and non-operative specialties (36.3% vs. 41.8%; *p* = 0.523; Fig. 3).

The perception that childcare was mainly provided by the female parent differed significantly across several groups. Men agreed or strongly agreed with this statement more frequently than women (81.5% vs. 52.8%; *p* = 0.002; Fig. 3). Agreement was also more frequent among physicians not working full-time than among full-time physicians (62.8% vs. 46.0%; *p* = 0.001) and among physicians in non-operative compared with operative specialties (61.3% vs. 50.6%; *p* = 0.014; Fig. 3).

Men more frequently agreed or strongly agreed that fathers commonly took parental leave than women (67.5% vs. 48.2%; *p* = 0.004; Fig. 3). Although the proportions agreeing or strongly agreeing were similar among full-time and non-full-time physicians (49.6% vs. 50.3%), the complete five-category response distributions differed significantly (*p* = 0.031; Fig. 3). No significant difference was observed between operative and non-operative specialties (48.8% vs. 51.1%; *p* = 0.098; Fig. 3).

A substantial majority of respondents expressed a desire for greater employer support regarding work–family balance. Agreement was similarly high among women and men (72.5% vs. 71.6%; *p* = 0.144; Fig. 3). Full-time physicians more frequently agreed or strongly agreed than physicians not working full-time (76.3% vs. 67.5%; *p* = 0.002), as did physicians in operative compared with non-operative specialties (77.2% vs. 67.1%; *p* = 0.011; Fig. 3).

In the aOR analysis, hospital employment was associated with greater difficulty balancing family responsibilities and work (aOR 2.44; *p* < 0.001; Table 5). Compared with physicians in leadership positions, resident physicians (aOR 2.45; *p* < 0.001) and board-certified physicians without leadership responsibility (aOR 2.02; *p* < 0.001; Table 5) had higher odds of reporting greater difficulty. Gender, work-time arrangement, and specialty group were not independently associated with this outcome (Table 5).

**Table 5:** Multivariable ordinal logistic regression analyses of difficulty balancing family responsibilities and work

Predictor	aOR (95% CI)	*p* value
Women vs. men	1.13 (0.75–1.70)	*0.572*
Full-time vs. non-full-time	1.16 (0.88–1.52)	*0.302*
Hospital vs. non-hospital	2.44 (1.74–3.43)	*< 0.001*
Non-operative vs. operative	0.80 (0.63–1.02)	*0.075*
Resident physicians vs. physicians in leadership positions	2.45 (1.66–3.62)	< 0.001
Board-certified physicians without leadership vs. physicians in leadership positions	2.02 (1.36–3.00)	*< 0.001*
No children vs. parents	0.40 (0.30–0.53)	*< 0.001*

The multivariable model included 897 participants. All predictors shown were included simultaneously. An aOR > 1 indicates higher odds of reporting a higher response category. Reference categories were men, non-full-time employment, non-hospital setting, operative specialty, physicians in leadership positions, and parents. The model was statistically significant (*p* < 0.001). For difficulty balancing family responsibilities and work, a higher category indicates greater difficulty

## Discussion

This study provides one of the largest contemporary assessments of work-time arrangements among physicians in Germany. Although full-time employment remained the most common arrangement, 43.2% of respondents were classified as not working full-time. Concerns about career disadvantages associated with alternative work-time arrangements were widespread. In the adjusted analyses, hospital-based physicians and early career physicians consistently reported less favorable outcomes across all five domains: satisfaction with their current work-time arrangement, employer support for and colleague acceptance of alternative arrangements, equal career opportunities for colleagues using these arrangements, and work–family balance.

### Study population and working-time patterns

The composition of the study sample differed from that of the overall physician workforce in Germany. Women represented 90.9% of the study population compared with 49.0% of physicians nationally, while 83.6% of participants worked in hospitals compared with 51.6% of the national physician workforce [1]. In 2022, the largest groups by recognized specialty in Germany were internal medicine (14.4%), general practice (10.6%), and surgery (9.7%), indicating that surgical specialties were more strongly represented in this study (32.4%) [1]. These differences should be considered when interpreting the findings, particularly regarding gender and specialty distribution.

In line with previous surveys, full-time employment was the most common work-time arrangement in our study, although its prevalence of 57% was lower than the 64% reported in the 2024 survey of the Marburger Bund [3]. Approximately 43% of respondents were not working full-time, a proportion that is likely influenced by the high share of female participants in our cohort and is consistent with earlier findings demonstrating a higher prevalence of part-time work among female physicians [3]. Part-time physicians in Germany report average weekly working hours of nearly 39 h, corresponding to a standard full-time workload in many non-medical professions [3]. By comparison, physicians employed full-time worked an average of 55 h per week, and 24% reported working at least 60 h per week [3]. These findings illustrate the substantial discrepancy that may exist between part-time employment status and physicians’ actual working hours when overtime and additional duties are considered [3]. Under the general provisions of the German Working Time Act (Arbeitszeitgesetz, ArbZG), working time is generally limited to eight hours per working day, extendable to ten hours subject to compensatory periods, and a minimum rest period of 11 h applies; specific exceptions exist for hospitals and under collective agreements (§§ 3, 5, and 7 ArbZG).

### Work-time arrangements and satisfaction

Part-time employment is frequently associated with improved work–life balance, work-family balance and higher quality of life [2, 8–10]. In our study, physicians not working full-time more frequently agreed or strongly agreed that they were satisfied with their current work-time arrangement than full-time physicians (52.8% vs. 45.3%). Furthermore, hospital employment was independently associated with lower satisfaction with the current work-time arrangement (aOR 0.33). Hospital work is frequently characterized by shift work, on-call duties, overtime, limited schedule predictability, and restricted individual control over working hours [3, 11]. These conditions may reduce satisfaction regardless of whether physicians are formally employed full-time or part-time. In contrast, employment outside the hospital setting may offer greater autonomy and predictability. Early career physicians had substantially lower odds of reporting greater satisfaction with their work-time arrangements. Greater autonomy and control associated with physician leadership may contribute to this difference, as autonomy has been identified as an important pathway linking leadership with physician well-being [12, 13].

The potential effects of the working environment and working conditions on physicians’ psychosocial health have received increasing attention, particularly in specialties traditionally characterized by full-time employment and hierarchical structures, such as surgery, which also represented the largest specialty group in our cohort [14–19]. High workload, long working hours, and work intensification have repeatedly been identified as key contributors to burnout, depressive symptoms, and reduced job satisfaction [3, 20–25]. Moreover, perceived adverse working conditions have been shown to correlate not only with physicians’ subjective well-being, but also with objective workload measures and patient-related outcomes, underscoring the systemic relevance of work-time arrangements that support work–life balance beyond their effects on individual health [26]. Further studies should investigate the precise relationship between satisfaction with the work-time arrangement, actual weekly working hours, and, ultimately, work–family balance.

### Parenthood, gendered care responsibilities, and career consequences

As the proportion of women in medicine increases and partnership models evolve toward shared parental responsibilities, requiring working conditions that enable both women and men to combine family life with career development [26, 27]. One way to meet this need is to provide opportunities for alternative work-time arrangements, such as part-time employment, which has been associated with lower levels of work–family conflict in previous studies [2]. Therefore, part-time employment is common among parents who are both physicians, with one parent reducing their working hours to approximately 30 h per week on average [27]. Among physicians, women are more likely to assume primary responsibility for family and care duties, which increases the likelihood of part-time employment by up to twenty-fold when children are present [28, 29]. Thus, family formation is particularly in dual-physician partnerships frequently associated with pronounced work–life conflicts, that negatively affect career progression and contributes to the gender pay gap [2, 10, 27, 30]. These dynamics are particularly pronounced during the third and fourth decades of life, when career advancement often coincides with family formation. During this phase, the gender care gap increases to over 100% [28]. This unequal distribution of care responsibilities contributes to delayed career progression, reduced access to academic opportunities, and persistent gender disparities in leadership positions [31–35].

These findings are in line with national and international studies across multiple specialties, including rheumatology, urology, and internal medicine, which consistently demonstrate reduced career opportunities for part-time physicians [10, 28, 29]. Previous studies indicate that physicians working part-time experience disadvantages in career advancement, with women being particularly affected due to their higher likelihood of working part-time and therefore more frequently exposed to these negative career consequences [9, 28, 29]. These mechanisms contribute to the so-called “female career dip”. In Germany, the proportion of female heads of university surgical departments has remained at approximately 5% for years [36–39]. This persistent underrepresentation of women in leadership positions illustrates the broader structural context in which concerns about career disadvantages associated with reducing work hours arise.

### Work–family balance and organizational responsibility

The ability to balance work and family life is already a key consideration for medical students and influences their subsequent choice of specialty. Indeed, up to 80% consider part-time work and regulated working hours important when choosing their future career [6, 40].

A 2025 cross-sectional survey of 676 medical students from 32 medical faculties in Germany and Austria found that interest in the specialty was the most important factor influencing specialty choice, followed by the work environment and culture, compatibility with family life, and work–life balance. Work environment and culture, compatibility with family life, and work–life balance received mean ratings exceeding four points on a five-point Likert scale. Female students rated each of these factors significantly higher than male students. Although these findings relate to medical students and cannot be directly compared with our sample of practicing physicians, they indicate a potential mismatch between the expectations of the future medical workforce and the working conditions experienced by physicians in the present study.

In our study, more than half of the respondents reported difficulties balancing work and family responsibilities, particularly hospital-based physicians (aOR 2.4) and resident physicians (aOR 2.5). By contrast, gender, working-time arrangement, and specialty group were not independently associated with such difficulties. Although physicians in operative specialties reported greater difficulty in the unadjusted comparison, this association was no longer statistically significant after adjustment. Nevertheless, previous studies of physician parents have found that work–family compatibility, particularly in surgical specialties, is moderate at best [27]. Further representative studies should specifically examine whether physicians in operative specialties experience greater difficulties balancing work and family responsibilities than those in non-operative specialties.

Nevertheless, difficulties in achieving work–family balance predominantly affect early-career hospital-based physicians—a group that, given that women account for 70% of medical students in Germany, consists primarily of women [41, 42]. Younger age and female gender have been identified as relevant risk factors for burnout and depression among physicians [2, 43], underscoring the vulnerability of early-career female physicians who frequently navigate competing demands in professional and private life. At the same time, reducing working hours may raise concerns about potential career setbacks, as reflected in the responses of participants in the present study. In the unadjusted comparisons, such concerns were most pronounced among women, full-time physicians, and physicians in operative specialties. Fear of negative career consequences was the most frequently reported reason for deciding against a desired reduction in working hours, cited by 58% of respondents. In addition, approximately two-thirds of participants perceived unequal career opportunities for colleagues using alternative work-time arrangements, with less favorable perceptions particularly among hospital-based and early-career physicians. These findings suggest that anticipated career disadvantages remain a major barrier to reduce working hours.

### The role of employers and organizational responsibility

The findings of this study highlight the central role of employers in shaping work-time arrangements and their acceptance within healthcare organizations. Fewer than half of the participants reported that their employer supported alternative work-time arrangements, whereas the vast majority reported that employees working under such arrangements did not receive the same level of acceptance as full-time employees. Not least because of the perceived unequal distribution of the workload, conflicts can arise at both the organizational and interpersonal levels. Together, these findings may indicate that flexible arrangements are often implemented individually rather than embedded within comprehensive organizational strategies addressing staffing, workload distribution, and scheduling.

Given that physicians typically enter parenthood during critical phases of career development, the provision of childcare compatible with clinical working hours constitutes a cornerstone of workforce retention and job satisfaction [44, 45]. Only approximately one-quarter of respondents reported having access to childcare with hours compatible with their work schedules. At the same time, more than 75% of the respondents indicated that they would use an employer-operated childcare facility. These findings highlight the potential of workplace-based childcare services to improve job satisfaction and work–family balance. At the macro level, targeted policy measures could further support the reconciliation of work and family life. Such measures include expanding reliable, publicly funded childcare services and reducing financial barriers to access. In Germany, for example, childcare is generally free of parental fees in several federal states, e.g. Berlin. Similarly, evidence from Norway suggests that widely available childcare, generous parental leave policies, and opportunities to reduce working hours may facilitate work–family balance among physicians [46].

Previous studies have emphasized the need for continuous monitoring of working conditions and mental health outcomes among physicians [19]. Nevertheless, rigid workflows, outdated organizational structures, and high administrative demands continue to impede the implementation of flexible work-time arrangements, particularly in hospital settings [47]. Digitalization and the integration of artificial intelligence (AI) offer substantial potential to improve efficiency, reduce administrative workload, and facilitate flexible working arrangements, including the possibility of performing administrative tasks remotely [47, 48]. AI-based tools may further optimize scheduling, and streamline documentation, thereby potentially freeing up time for direct patient care and enabling more adaptable work structures [48].

### Implications for workforce retention

Against the background of physician shortages and increasing patient volumes, the retention of qualified medical staff is of critical importance. Difficulties balancing work and family predominantly affect hospital-based physicians and those at earlier career stages. Strategies designed to support work–family balance through alternative work-time arrangements should therefore not be accompanied by diminished career prospects. Although professional competence is not inherently linked to the number of contracted working hours, structural career progression often remains tied to traditional full-time norms [6]. Addressing this discrepancy is essential to ensure equal career opportunities in a medical workforce that is becoming increasingly diverse with regard to gender roles, family models, and life-course expectations. Family-friendly and flexible working conditions may play a key role in improving job satisfaction and supporting long-term workforce retention. Flexible work-time arrangements, including part-time models and temporary adjustments to working hours, therefore represent a strategic investment in physicians who may remain in the workforce for several decades [3, 5].

### Limitations

Several limitations should be considered when interpreting the findings of this study.

The questionnaire was developed specifically for this survey and did not include validated instruments measuring work–family or family–work conflict. This limits the assessment of construct validity and the comparability of the findings with studies using established psychometric scales. Alternative work-time arrangements were not defined a priori using standardized operational criteria. Terms such as remote work, flexitime, and job-sharing therefore reflected participants’ own understanding and may have been interpreted heterogeneously.

Recruitment via the mailing list of the German Medical Women’s Association and through professional, personal, and social networks may have introduced selection bias. Women, hospital-based physicians, and physicians working in surgical specialties were markedly overrepresented compared with the overall physician workforce in Germany. Also, recruitment through the German Medical Women’s Association likely contributed to the high proportion of female participants, while dissemination through the authors’ professional networks may have contributed to the overrepresentation of surgical specialties. In addition, physicians with a particular interest in work-time arrangements or family-friendly working conditions may have been more likely to participate.

Because the total number of individuals who received or encountered the survey invitation could not be determined, a valid response rate could not be calculated, and potential non-response bias could not be assessed. The sample should therefore not be considered representative of the entire physician workforce in Germany, which limits the generalizability of the findings.

Data were collected between 2022 and 2023. While this temporal aspect should be acknowledged, structural conditions within German hospitals and healthcare institutions have undergone little fundamental change during this period. Ongoing debates on physician shortages, burnout, and work–life balance, as well as recent workforce surveys, indicate that the challenges identified in this study remain highly relevant.

## Conclusions

Overall, the findings indicate that work-time arrangements in medicine remain largely aligned with traditional full-time structures, despite growing dissatisfaction and changing expectations among physicians. Part-time and alternative work-time arrangements are still widely perceived as career-limiting, which constitutes a major barrier to their broader implementation.

Ensuring equal career opportunities for physicians who require or prefer family-friendly working conditions is essential for gender equity, workforce sustainability, and organizational resilience. As care responsibilities and expectations of work–life balance increasingly affect both women and men, addressing structural barriers to flexible work-time arrangements is no longer a niche issue, but a central challenge for healthcare systems facing demographic and societal change.

## Supplementary Information

Below is the link to the electronic supplementary material.

Supplementary Material 1

## Data Availability

The datasets used and analysed during the current study are available from the corresponding author on reasonable request.

## Abbreviations

AI: Artificial intelligence
aOR: Adjusted odds ratio
ArbZG: German Working Time Act (Arbeitszeitgesetz)
CI: Confidence interval
DÄB: German Medical Women’s Association (Deutscher Ärztinnenbund)
SPSS: Statistical Package for the Social Sciences
vs: versus
