# Supplementary material for: Work-time arrangements, career progression, and job satisfaction among physicians in Germany: a cross-sectional survey
Source: BMC Health Serv Res. 2026 Oct 21;26:1441. doi: 10.1186/s12913-026-15756-9 (PMC13688778; doi:10.1186/s12913-026-15756-9)
Supplement: Supplementary file 1 [file 12913_2026_15756_MOESM1_ESM.docx]

ADDITIONAL FILE 1:

**Survey Questionnaire: Family-Friendliness of Working Conditions in Medicine**

The questionnaire was originally developed in German and translated into English for reporting purposes.

***Section 1: General Information***

Thank you for participating in this survey on work–family balance in medicine.

This questionnaire is aimed at physicians working in both inpatient and outpatient settings who face the daily challenge of balancing professional and family responsibilities.

All responses are anonymous. Completion of the questionnaire takes approximately 5 minutes.

* Mandatory questions

**1. Gender***

Female/ Male/ Diverse

**2. Age group***

- < 25
- 25–< 30
- 30– <35
- 35– <40
- 40–45
- >45

**3. Medical specialty* (multiple choice)**

- Surgery
- Internal Medicine
- Anesthesiology
- Pediatrics
- Urology
- Gynecology and Obstetrics
- Dermatology
- Radiology
- Neurology
- Otorhinolaryngology (ENT)
- Ophthalmology
- General Practice
- Orthopedics
- Other: _________

**4. Do you have children requiring care?***

- No children
- 1
- 2
- 3
- More than 3

**5. Current work setting*** (multiple choice)

- Hospital
- Private practice
- Research
- Self-employed
- Other: _________

**6. Is your workplace located in an urban or rural area?***

- Urban
- Rural

**7. Current position***

- Resident physician
- Specialist (board-certified physician)
- Senior physician (consultant)
- Senior physician in a leading position
- Head of department (chief physician)

**8. How would you describe your current working time model?*** (multiple choice)

- Full-time
- Part-time
- Home office
- Job sharing
- Flexible working hours
- Other

**8.1 If “Other”:**

Please specify: _________

**9. I am satisfied with my current working time model.***

(Likert scale: 1 = strongly disagree to 5 = strongly agree)

**10. Balancing family responsibilities and work is currently a problem for me.***

(Likert scale: 1 = strongly disagree to 5 = strongly agree)

**11. Alternative working time models are supported by my employer.***

(Likert scale: 1 = strongly disagree to 5 = strongly agree)

**12. Colleagues using alternative working time models are as accepted as full-time staff in my department.***

(Likert scale: 1 = strongly disagree to 5 = strongly agree)

**13. Colleagues using alternative working time models have the same career opportunities as full-time staff.***

(Likert scale: 1 = strongly disagree to 5 = strongly agree)

**14. Do alternative working time models result in a higher workload for full-time staff?***

(Likert scale: 1 = strongly disagree to 5 = strongly agree)

**15. In my professional environment, there are colleagues in leadership positions who successfully combine career and family.***

(Likert scale: 1 = strongly disagree to 5 = strongly agree)

**16. In my department, pregnant employees can continue working on request, possibly in alternative roles (e.g., home office).***

(Likert scale: 1 = strongly disagree to 5 = strongly agree)

**17. The distribution of workload (e.g., weekend duties) for part-time employees in my department is fair.***

(Likert scale: 1 = strongly disagree to 5 = strongly agree)

**18. My department responds flexibly to sudden family obligations.***

(Likert scale: 1 = strongly disagree to 5 = strongly agree)

**19. I would like more support from my employer regarding work–family balance.***

(Likert scale: 1 = strongly disagree to 5 = strongly agree)

**20. In my department, it is now common for fathers to take parental leave.**

(Likert scale: 1 = strongly disagree to 5 = strongly agree)

**21. Imagine you would like to reduce your working hours but decide against it. Why?** (multiple choice)

- My employer does not accept alternative working time models
- Staff shortage
- Fear of negative career consequences
- Competitive pressure
- Concern about others’ opinions

**22. Do employees have external access to patient data (e.g., VPN)?***

- Yes, only physicians in leadership positions
- Yes, all employees
- No
- Unknown

**23. Is your department certified as family-friendly (e.g., “audit berufundfamilie”)?**

- Yes
- No
- I have never heard of this certification
- Unknown

***Section 2: Support Structures and Childcare***

**24. Are there specific support services for employees with families in your department? If yes, which?** (multiple choice)

- No, none
- Support in finding childcare places
- Holiday programs for children
- Counseling (e.g., equal opportunities officer, career counseling)
- VPN access for home office
- Other

**21.1 If “Other”:**

Please specify: _________

**25. Do you have specific ideas on how to improve work–family balance?**

(Open-ended response)

**Childcare**

The following section focuses on childcare and your current situation.

If not applicable, you may skip this section.

**26. What form of childcare do you use? (multiple choice)**

- Partner provides childcare at home
- Daycare / kindergarten
- Childminder (daycare provider)
- Support from family/friends/neighbors
- Au pair / nanny
- After-school care
- Other

**26.1 If “Other”:**

Please specify: _________

**27. What challenges do you face regarding childcare? (multiple choice)**

- Opening hours of childcare facilities
- Distance between workplace, home, and childcare
- Financial burden
- Lack of childcare places
- Quality of care
- Other

**27.1 If “Other”:**

Please specify: _________

**28. Are you a single parent?**

- Yes
- No

**29. Is employer-organized childcare available?**

- Yes, with working-hour-compatible opening times
- Yes, but opening times are not compatible
- No

**30. Would you use employer-organized childcare?**

- Yes
- No

**31. How do you organize work and childcare responsibilities? (multiple choice)**

- Both parents work full-time
- Both parents have reduced working hours
- One parent stays at home, the other works as before
- Shared parental leave
- Alternating arrangements
- Not applicable (single parent)

**32. It would make my life easier if I did not have to strictly adhere to core working hours (except in shift work).**

(Likert scale: 1 = strongly disagree to 5 = strongly agree)

**33. In my household, childcare is mainly provided by the female parent.**

(Likert scale: 1 = strongly disagree to 5 = strongly agree)

**34. I am afraid of negative career consequences if I reduce my working hours to spend more time with my family.**

(Likert scale: 1 = strongly disagree to 5 = strongly agree)
